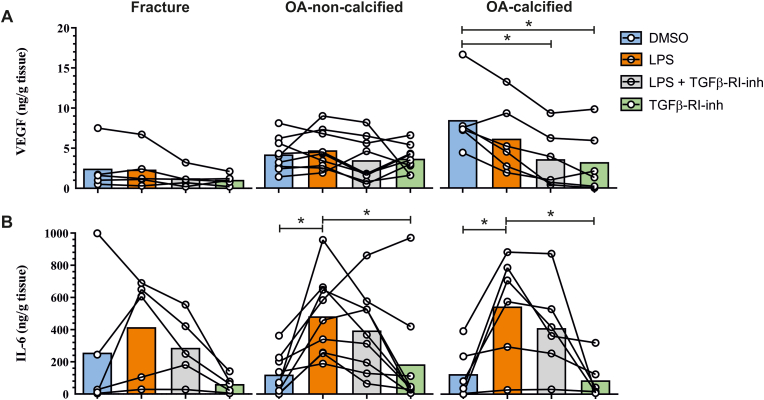# Elevated secretion of pro-collagen I-alpha and vascular endothelial growth factor as biomarkers of acetabular labrum degeneration and calcification in hip osteoarthritis: An explant study

**DOI:** 10.1016/j.jot.2023.08.007

**Published:** 2023-12-26

**Authors:** Alexander Antoniadis, Julien Wegrzyn, Patrick Omoumi, Léa Loisay, Thomas Hügle, Jeroen Geurts

**Affiliations:** a Department of Orthopedics, Lausanne University Hospital and University of Lausanne, Switzerland; b Department of Diagnostic and Interventional Radiology, Lausanne University Hospital and University of Lausanne, Switzerland; c Department of Rheumatology, Lausanne University Hospital and University of Lausanne, Switzerland

**Keywords:** Acetabular labrum, Biomarker, Explant model, Hip osteoarthritis, Inflammation, Pathologic calcification

## Abstract

**Background:**

Hip osteoarthritis (OA) involves structural degeneration of different joint compartments, including femoral head cartilage, periarticular ligaments and the acetabular labrum. However, the molecular mechanisms underlying labrum degeneration in hip OA remain poorly understood.

**Aim:**

To assess secretion of putative biomarkers for OA from explanted human labrum tissues under basal and inflammatory conditions and to determine whether these could differentiate between OA and calcification status compared to fracture controls.

**Methods:**

Intact labrum specimens were collected from patients undergoing joint arthroplasty for primary hip OA (*n* ​= ​15, mean age 70) or non-OA femoral neck fracture (*n* ​= ​5, mean age 64). Tissues were dissected in equal-sized samples and explanted for one week. To mimic activation of inflammatory signaling by endogenous damage-associated molecular patterns (DAMP) tissue were stimulated with a toll-like receptor 4 (TLR4) agonist (1 ​μg/mL LPS). The involvement of transforming growth factor-beta (TGF-beta) signaling was evaluated by treatment with a TGF-beta type 1 receptor inhibitor (10 ​μM SB-505124). Secretion of aggrecan (ACAN), pro-collagen-I alpha (Pro-Col-Iα), cartilage oligomeric matrix protein (COMP), interleukin-6 (IL-6) and vascular endothelial growth factor (VEGF) was assessed by enzyme-linked immunosorbent assay (ELISA). Labrum calcification was evaluated by 3D whole mount fluorescent microscopy of ethyl cinnamate-based optically cleared tissues stained with Alcian blue/Alizarin red.

**Results:**

Whole mount microscopy revealed non-OA fracture controls were non-calcified, whereas six OA labra (40%) were partially calcified or ossified. Basal secretion of Pro-Col-Iα and VEGF was increased four-fold in OA versus non-OA labra. Pro-Col-Iα levels were correlated with those of VEGF (*r* ​= ​0.65) and COMP (*r* ​= ​0.54). Stimulation of DAMP signaling through TLR4 affected secretion of IL-6, VEGF, COMP and Pro-Col-Iα, with distinct responses between non-OA and OA tissues. Inhibition of TGF-beta signaling specifically reduced elevated secretion of Pro-Col- Iα and VEGF in calcified OA labrum.

**Conclusions:**

Secretion of the putative OA biomarkers Pro-Col-Iα and VEGF is elevated in degenerated human acetabular labrum and may serve as indicators of OA and calcification status. Secretion of both factors was partially regulated by TGF-beta signaling in calcified OA labrum tissues.

*The Translational potential of this article:*

Our findings suggest that a biomarker panel consisting of Pro-Col-Iα/VEGF/COMP may be valuable for assessing subradiographic labrum degeneration and calcification in hip OA. Targeting TGF-beta signaling may offer a means to reduce vascular invasion and fibrosis in acetabular labrum tissue.

## Graphical abstract

**Figure:**
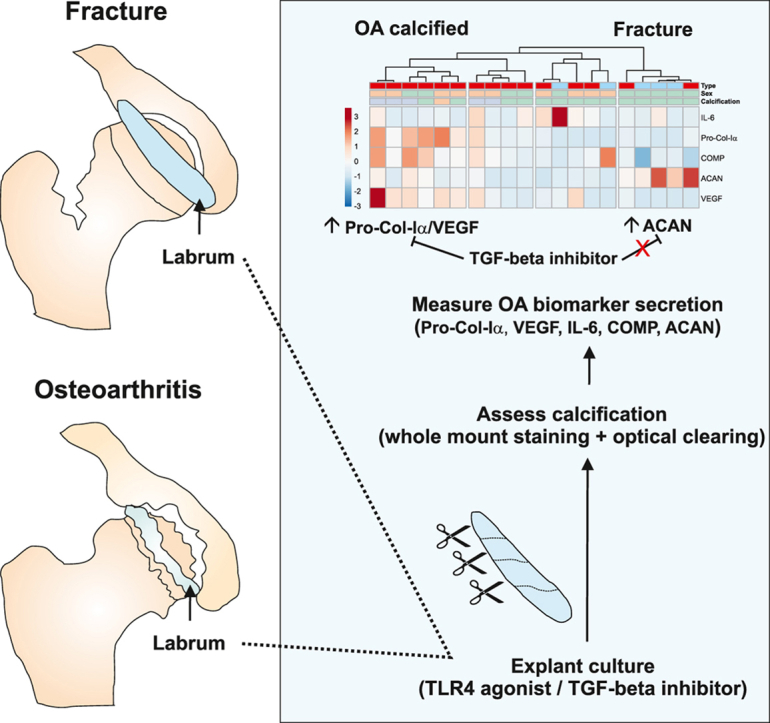

## 1 Introduction

Hip osteoarthritis (OA) affects several compartments of the joint including hyaline cartilage, subchondral bone, the joint capsule as well as periarticular ligaments and fibrocartilage [1]. The acetabular labrum is composed of ring-form fibrocartilage and plays a fundamental role in hip joint stability and articular cartilage homeostasis by regulating joint lubrication, load distribution, proprioception and mechanotransduction. All labral portions are in contact with the acetabular hyaline cartilage and thus articulate with the analogous femoral head articular cartilage. Cadaver studies of intact labrum tissues of aging individuals have demonstrated that the acetabular labrum is both vascularized and innervated [2]. Degeneration and structural damage of labrum tissue is believed to play pivotal roles in degenerative hip conditions, such as femoroacetabular impingement (FAI) and primary hip OA [3]. Labral tears are associated with hip osteophytes, but it is unclear how these two features are causally related [4].

Degenerated labrum tissues from OA hip joints have undergone extensive characterization through histopathological and transcriptomic analyses [[5], [6], [7], [8], [9], [10], [11], [12], [13]]. Histological studies have revealed a range of pathological alterations including chondrocyte apoptosis, macrophage infiltration, vascular proliferation, matrix destruction and intralabral calcifications [5,7,10]. Labral calcification were found to be highly prevalent (100%) in a cohort of 80 patients, and the extent of calcification was associated with the degree of cartilage damage regardless of age [9]. Calcifications in end-stage hip OA were negatively correlated with hip function and positively correlated with pain scores [10]. Transcriptomic analyses have unveiled distinct gene expression profiles between primary labral fibro-chondrocytes and articular chondrocytes in both human and bovine species [6,8,11,13]. *In vitro* studies involving mechanical loading or cytokine stimulation of labral cells have demonstrated upregulation of extracellular matrix genes such as aggrecan (ACAN), type I collagen (COL1A), cartilage oligomeric matrix protein (COMP) and interleukin-6 (IL-6) [6,13]. Gene pathway enrichment studies conducted on labrum cells from healthy and OA tissues have suggested differential activation of pro-inflammatory, pro-angiogenic and transforming growth factor-beta (TGF-beta) pathways [8,11]. Importantly, proteomic studies have identified differential protein levels of the aforementioned genes in the synovial fluid of hip and knee OA patients [14,15]. Notably, ACAN and Pro-Col-Iα exhibited differential expression between early OA and control samples [15].

In this study, we sought to characterize secretion of selected putative OA biomarkers (ACAN, Pro-Col-Iα, COMP, IL-6, VEGF) by anatomically intact acetabular labrum tissues exhibiting different OA and calcification status. Additionally, we assessed whether these proteins could be regulated through the activation of damage-associated molecular patterns (DAMP) signaling, which plays a crucial role in several inflammatory pathways in the OA joint [16]. Lastly, we examined the potential involvement of TGF-beta signaling in the secretion of these biomarkers.

## 2 Methods

### 2.1 Collection of clinical specimens and clinico-radiological data

The study was reviewed and approved by the local ethical committee. Acetabular labrum tissue was harvested from consented patients undergoing total hip arthroplasty for primary hip OA (*n* ​= ​15) or femoral neck fracture (*n* ​= ​5). Exclusion criteria were secondary hip OA, metabolic bone disorders and prior OA diagnosis in fracture cases. Samples were collected in sterile saline and stored at 4 ​°C until further processing. Prior to the joint replacement procedure, anteroposterior pelvic radiographs were obtained for all patients prior to the joint replacement procedure. One musculoskeletal radiologist with 12 years of experience reported the presence or absence of acetabular rim calcifications or ossification on radiographs. Acetabular rim calcifications were defined as opacities projecting laterally in close vicinity but detached from the acetabular rim, while acetabular rim ossifications were defined using the double-rim sign [17,18]. Clinical and radiographic data are summarized in Table 1.

**Table 1:** Patient demographics, radiological assessments, and levels of extracellular matrix protein and pro-inflammatory cytokine secretion by explanted labra from primary hip OA patients and fracture controls.

Parameter	Hip OA (n ​= ​15)	Fracture (n ​= ​5)	P-value
Age, years	70 ​± ​11	64 ​± ​6	0.28
Sex, no. female (%)	6 (40%)	4 (80%)	0.30
Acetabular rim ossification, no. (%)	12 (80%)	3 (60%)	0.56
Radiographic labrum calcification, no. (%)	1 (7%)	0 (0%)	0.99
**Basal secretion**			
Pro-Col-Iα, ng/g (range)	410 (47–1816)	105 (22–218)	**0.005**
COMP, ng/g (range)	5002 (2067–10019)	3796 (926–10645)	0.13
ACAN, ng/g (range)	4 (0.3–25)	10 (2–24)	0.50
VEGF, ng/g (range)	5.6 (1.4–17)	1.6 (0.5–8)	**0.005**
IL-6, ng/g (range)	72 (0–391)	27 (4–1000)	0.86

### 2.2 Explant culture of labrum tissue

Explant culture of human labrum tissues was adapted from a method developed previously for osteochondral joint tissues [19]. Clinical specimens were processed within 2 ​h post-surgery and dissected with a scalpel into equal-sized samples (200–400 ​mg wet weight). Dissected tissues were cultured in 8 ​mL αMEM (10% FBS, 10 ​mM HEPES, 4 ​mM l-glutamine) for 1 ​week ​at 37 ​°C in a humidified atmosphere containing 5% CO_2_. Specimens were treated with 1 ​μg/mL LPS (*Escherichia coli* O111:B4, L2630 Sigma–Aldrich) in the presence of a TGF-beta receptor type I inhibitor (10 ​μM SB-505124) or DMSO vehicle (0.00075% v/v) at day 1 and 4. Conditioned medium was collected at day 7 and stored at −80 ​°C until further analysis. Labrum tissues were fixed in formalin for 48 ​h at 4 ​°C.

### 2.3 Enzyme-linked immunosorbent assay (ELISA)

Secreted protein levels were assessed by commercial ELISA kits for Pro-Col-Iα (Abcam, ab216064, sample dilution 1:100), aggrecan (RayBiotech, ELH-ACAN-1, sample dilution 1:3), COMP (Sino Biological, SEK10173, sample dilution 1:500), vascular endothelial growth factor (VEGF)-121 (Sino Biological, SEK10008, sample dilution 1:2) and IL-6 (Invitrogen, 88-7066-86, sample dilution 1:400). Protein levels were normalized to wet weight of explanted samples and expressed as ng/g tissue.

### 2.4 Whole-mount microscopy and optical clearing

Formalin-fixed labrum samples were dehydrated in two volumes 95% ethanol overnight at room temperature. Proteoglycans were stained overnight with 0.03% w/v Alcian blue 8GX in 80% ethanol/20% glacial acetic acid. Samples were destained in two volumes 70% ethanol before staining overnight with 0.005% w/v Alizarin red S in 1% KOH. Samples were destained in two volumes 1% KOH and dehydrated in ethanol. Double-stained labrum tissues were cleared in ethyl cinnamate. Brightfield and fluorescence imaging (Texas red bandpass filter, ex: 539 ​nm/em 630 ​nm) was performed on a Leica M205-FA stereomicroscope. Images were captured at magnification 20× and merged in Leica Application Suite X (Leica Microsystems CMS GmbH, version 3.0.14.23224).

### 2.5 Statistical analysis

Hierarchical clustering of normalized protein expression was performed in ClustVis 1.0 [20] using average linkage and correlation distance. Statistical analyses were performed using GraphPad Prism (v9.3, Graphpad Software Inc., San Diego, CA, USA). Group-wise comparisons of normally distributed data (Pro-Col-Iα, IL-6, COMP) were performed by ratio paired *t*-test and repeated measures one-way ANOVA with Tukey's multiple comparison test. For non-normally distributed data (VEGF, ACAN) Mann Whitney test and Friedman's test with Dunn's multiple comparison test. Spearman rank correlation was used for correlation analyses. P-values less than 0.05 were considered significant.

## 3 Results

### 3.1 Elevated secretion of Pro-Col-Iα and VEGF in labrum tissues from hip OA compared with fracture controls

The secretion of extracellular matrix proteins and pro-inflammatory cytokines was assessed in conditioned medium from labrum tissues that underwent one-week explant culture (Table 1). Comparing OA labrum samples with fracture controls, both Pro-Col-Iα and VEGF levels were found to be approximately fourfold higher in OA samples. Secretion levels of cartilaginous extracellular matrix proteins COMP and ACAN and IL-6 were similar in OA specimens and non-OA controls.

### 3.2 Whole mount imaging of optically-cleared tissue using Alizarin red fluorescence reveals labrum calcification status

Following *ex vivo* culture, explanted tissues were subjected to sequential whole mount staining with Alcian blue and Alizarin red staining to visualize proteoglycans and calcifications, respectively. Ethyl cinnamate-based tissue clearing facilitated 3D stereomicroscopy analysis of intact tissues (Fig. 1). Labrum tissues from fracture controls did not exhibit any focal calcifications, whereas six out of fifteen labra from OA patients displayed focal calcifications (*n* ​= ​5) or complete ossification (*n* ​= ​1). Alcian blue staining patterns appeared uniform and did not indicate significant proteoglycan loss in the presence or absence of calcification. While labrum calcifications were readily detectable through microscopic analyses, radiological assessment identified labrum calcification in only one case (Table 1). In contrast, ossification of the acetabular rim adjacent to the labrum was a common radiological feature, with no significant differences between groups (3/6 calcified OA, 9/9 non-calcified OA, 3/5 fracture controls).

**Figure 1:**
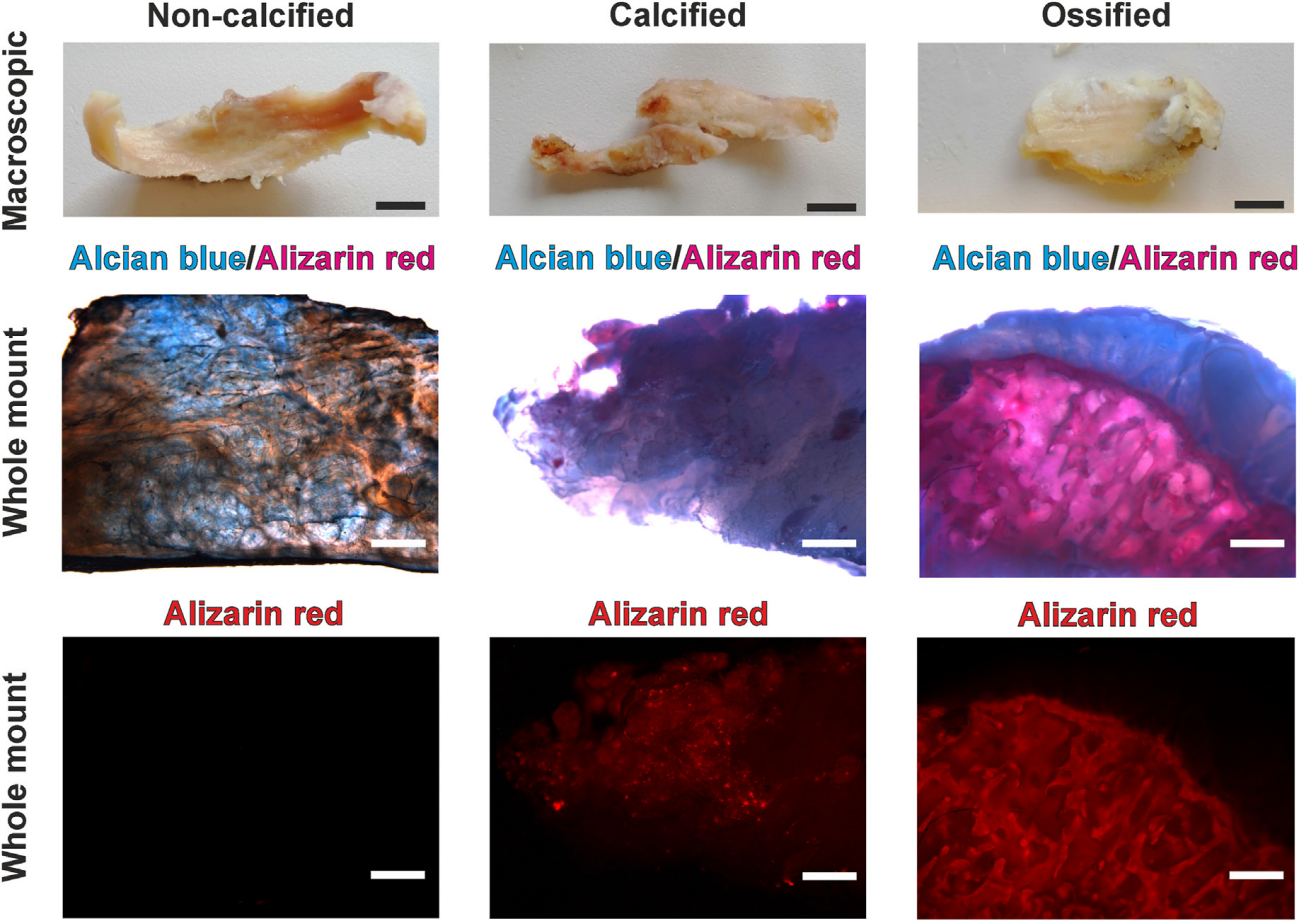
**Macroscopic and whole mount fluorescent imaging of acetabular labrum calcification using ethyl cinnamate-based optical clearing**. Representative images of fixed non-calcified (fracture, OA), calcified and ossified specimens prior to whole mount staining (upper panels). Following staining with Alcian blue/Alizarin red and optical clearing with ethyl cinnamate, homogeneous proteoglycan staining (blue) was observed without significant loss in OA specimens. Alizarin red staining highlighted calcifications (middle panels). Imaging of Alizarin red fluorescence in cleared tissues enabled clear localization of focal calcification in labrum tissue (lower panels). Fluorescent imaging was used for stratification of calcification status of labrum specimens. Scale bar: 1 ​cm for macroscopic images, 500 ​μm for whole mount microscopy. (For interpretation of the references to colour in this figure legend, the reader is referred to the Web version of this article.)

### 3.3 Biomarker panel can distinguish between calcified OA labrum and fracture controls

**Figure 2:**
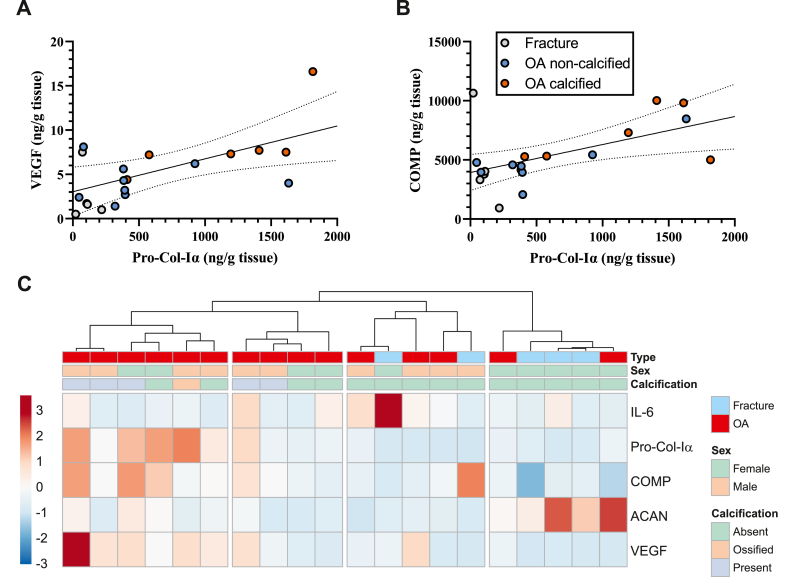
**Hierarchical clustering of baseline biomarker profiles in hip OA labra indicates distinct subgroups based on presence of calcification.** Scatter plot of Pro-Col-Iα levels versus VEGF **(A)** and COMP **(B)**. Regression line with 95% confidence intervals is depicted. **(C)** Biomarker levels were normalized to the average of the entire sample and depicted clusters were generated using average linkage clustering based on correlation distance. Compared to fracture controls, OA labra exhibited elevated expression of Pro-Col-Iα, COMP, and VEGF.

Secretion of Pro-Col-Iα under basal conditions was significantly correlated with that of VEGF (*r* ​= ​0.65, *p* ​= ​0.002) and COMP (*r* ​= ​0.54, *p* ​= ​0.013) (Fig. 2A and B). As regression plots suggested clustering of calcified OA and control groups, we investigated whether the biomarker panel could help distinguish the calcification status of labra. Using unsupervised hierarchical clustering of the five proteins, we identified two subgroups with clear separation of OA samples from fracture controls (Fig. 2C). By overlaying the clustering with microscopy assessment, we discovered that a subgroup exhibiting elevated levels of Pro-Col-Iα, COMP and VEGF secretion predominantly originated from calcified OA samples. Conversely, a cluster primarily composed of fracture controls demonstrated higher ACAN levels.

### 3.4 Mimicking DAMP signaling through TLR4 impacts secretion of Pro-Col-Iα, IL-6, VEGF and COMP

**Figure 3:**
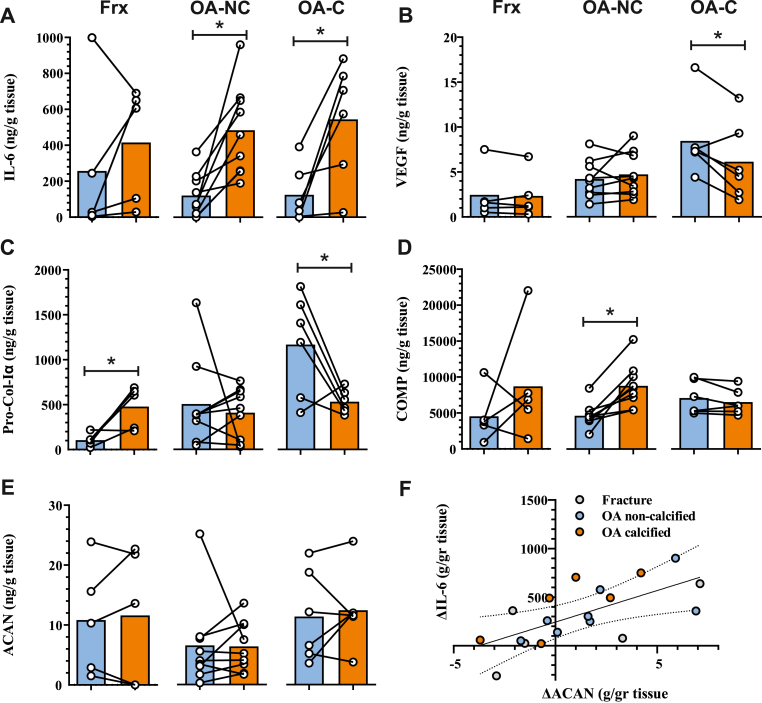
**Regulation of biomarker secretion through DAMP-mimicking activation of TLR4. (A**–**E)** Secretion of indicated biomarkers in fracture controls (Frx), non-calcified (OA-NC) and calcified OA (OA-C) labrum tissues treated with vehicle (blue) or LPS (orange). **(F)** Scatter plot of the relative change in IL-6 and ACAN secretion between vehicle and LPS treatments. ∗*P* ​< ​0.05 by Tukey's post-hoc test. (For interpretation of the references to colour in this figure legend, the reader is referred to the Web version of this article.)

Given that activation of toll-like receptor 4 (TLR4) signaling by endogenous DAMPs is known to play an important role in regulating pro-inflammatory cytokines in the OA joint [16], we investigated whether this signaling pathway could impact biomarker secretion in labrum tissue. Stimulation with LPS led to increased IL-6 secretion of in 13 out of 15 samples (Fig. 3A). Distinct responses to LPS were observed for the secretion of VEGF, Pro-Col-Iα and COMP among different labrum groups (Fig. 3B–D). Activation of TLR4 signaling notably reduced secretion of VEGF and Pro-Col-Iα in OA calcified labrum tissues only. Conversely, Pro-Col-Iα levels increased only in fracture controls and COMP levels were elevated only in non-calcified labrum. While the secretion of ACAN appeared unaffected by LPS treatment (Fig. 3E), there was a significant correlation (*r* ​= ​0.58, *p* ​= ​0.007) between the relative changes in IL-6 and ACAN secretion. Moreover, the effects of LPS treatment appeared to be independent of TGF-beta signaling (Supplementary Figs. 1 and 2).

### 3.5 TGF-beta signaling regulates elevated Pro-Col-Iα and VEGF secretion in calcified OA labrum tissues

**Figure 4:**
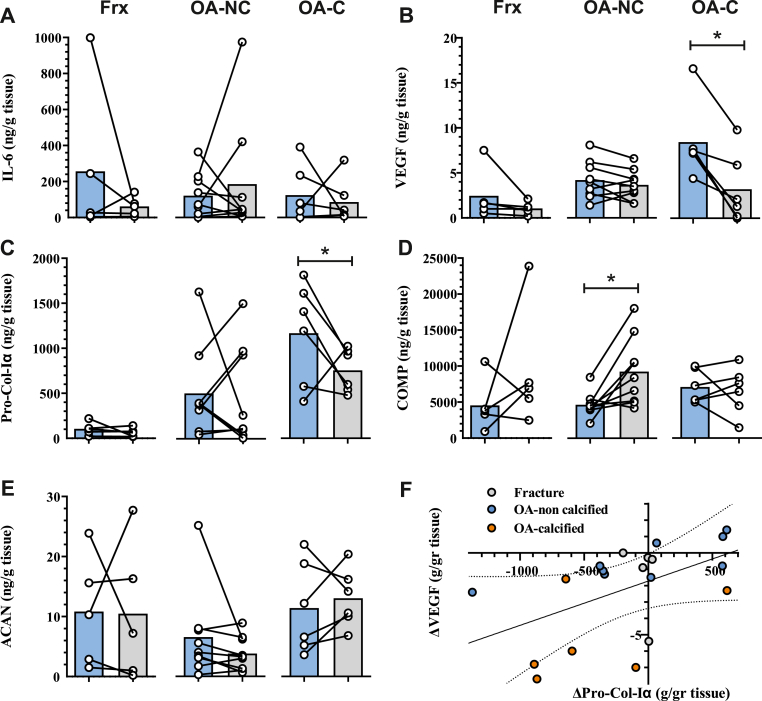
**Regulation of biomarker secretion through TGF-beta signaling. (A**–**E)** Secretion of indicated biomarkers in fracture controls (Frx), non-calcified (OA-NC) and calcified OA (OA-C) labrum tissues treated with vehicle (blue) or TGF-beta receptor I inhibitor (grey). **(F)** Scatter plot of the relative change in Pro-Col-Iα and VEGF secretion between vehicle and TGF-beta receptor I inhibitor treatments. ∗*P* ​< ​0.05 by Tukey's post-hoc test. (For interpretation of the references to colour in this figure legend, the reader is referred to the Web version of this article.)

Lastly, we investigated the involvement of TGF-beta signaling in regulating biomarker secretion under basal conditions. Treatment with a TGF-beta receptor I inhibitor resulted in a decrease in Pro-Col-Iα and VEGF secretion specifically in OA calcified labrum tissues (Fig. 4B and C). There was a significant correlation (*r* ​= ​0.59, *p* ​= ​0.006) between the relative changes in Pro-Col-Iα and VEGF secretion (Fig. 4F). Secretion of IL-6 and ACAN remained unaffected by inhibition of TGF-beta signaling (Fig. 4A,E). COMP levels were elevated only in non-calcified labrum (Fig. 4D) similar to the extent observed with LPS treatment (Supplementary Fig. 1).

## 4 Discussion

To the best of our knowledge, this study represents the first *ex vivo* characterization of intact acetabular labra in humans, shedding light on tissue responses and changes in this anatomical region. Our findings complement previous *in vitro* studies focusing on primary labrum cells [6,11,12,21]. Furthermore, we demonstrated the feasibility and efficiency of whole mount imaging combined with optical clearing for evaluating tissue calcification in human cartilaginous samples. In-depth analysis of selected putative OA biomarkers revealed distinct expression patterns of Pro-Col-Iα and VEGF, as well as their differential regulation by TLR4 and TGF-beta signaling, specifically in calcified labra compared to non-calcified OA specimens and fracture controls.

Consistent with the fibrocartilage nature, primary cells derived from OA patients expressed higher levels of collagen type I than type II when compared to articular cartilage [6]. *In vitro* studies have indicated that Pro-Col-Iα expression is negatively regulated by inflammation [6] and stimulated by mechanical stretching [12]. Interestingly, the upregulation of pro-osteogenic genes, including Pro-Col-Iα and VEGF, in response to mechanical stretching varied between fibrochondrocytes from mildly and severely degenerated labra [12]. Our *ex vivo* biomarker assessments corroborated these findings, suggesting that altered labrum biomechanics in OA may trigger secretion of pro-osteogenic factors. Additionally, VEGF levels were found to be significantly increased in hip OA compared to controls in proteomic profiling studies, whereas IL-6 levels showed no significant differences [14].

Responses to LPS treatment and inhibition of TGF-beta signaling in labral tissues exhibited noteworthy differences compared with our previous studies on articular cartilage [22] and osteochondral tissue explants [19]. While autocrine IL-6 pathological calcification of articular cartilage was promoted by autocrine IL-6 secretion [22], we did not detect elevated IL-6 tissue levels in OA labra or a significant association of Pro-Col-Iα and VEGF with IL-6. The observation that labrum calcification can proceed to tissue ossification, whereas cartilage calcification remains predominantly focal, supports the notion that both processes are regulated differently. We identified TGF-beta signaling as crucial regulator of Pro-Col-Iα and IL-6 tissue secretion in osteochondral tissues [19], while TGF-beta type I receptor inhibition affected secretion only in calcified OA labrum. Moreover, studies on primary labral fibrochondrocytes have suggested that BMP signaling might be primarily involved in regulating expression of pro-osteogenic genes [12]. In contrast, expression of extracellular matrix proteins in OA articular cartilage appeared to be regulated by TGF-beta and Wnt signaling [23]. These findings underscore the distinct tissue metabolism of labra [6,8,13], highlighting the need to consider them separately from our extensive knowledge of articular cartilage.

We made an intriguing observation regarding the high secretion of COMP by labral tissues and its positive correlation with pro-osteogenic factors Pro-Col-Iα and VEGF. This finding gains significance as a rare genotype with a missense mutation in COMP has been strongly linked to total joint replacement in hip OA [24]. Additionally, COMP has shown potential as a biomarker to distinguish e between control individuals and patients with hip lesions and femoroacetabular impingement [25]. Considering these findings, we propose that when studying the elevated risk of hip OA associated with COMP genotypes, the involvement of acetabular labrum should be taken into account alongside hip articular cartilage.

One important question that remains somewhat unclear is the clinical relevance of labrum pathology, particularly with regard to calcifications, in the diagnosis and treatment of hip OA. The presence of labral pathology, predominantly labral tears, was found in as high as 40% of a cohort consisting of young asymptomatic adult volunteers [26]. The prevalence increased to 86% in a cohort of symptomatic hip patients, primarily associated with the size of acetabular and femoral head osteophytes, rather than subchondral joint sclerosis or minimum joint space [4]. Notably, combined excision of labral calcifications, labral tear repair, and osteoplasty resulted in significant clinical and functional improvement in patients with femoroacetabular impingement syndrome up to two years following arthroscopy [27]. It is worth mentioning that the presence of labral calcification in histological analysis showed poor correlation with its detection on radiography. This discrepancy is likely due to the fact that radiography is not the most accurate imaging modality for detecting calcifications. In a study involving 3228 histologically confirmed calcified menisci, its sensitivity was only 35.3% [28]. Cross-sectional imaging methods such as computed tomography, which offer three-dimensional visualization, perform better in this regard [29,30]. Conversely, acetabular rim ossifications were commonly found in both fracture controls and OA samples, consistent with a previous report using computed tomography, which showed that acetabular rim ossifications are highly prevalent in asymptomatic individuals [18]. It is important to note that these ossifications are located immediately adjacent to the acetabular rim, while labral samples were mostly collected from the non-ossified portion of the labrum.

There are several limitations to our study related to the methodology employed. Firstly, our was based on putative biomarkers reported in literature rather than unbiased proteomic analysis. Conducting such comprehensive analyses would be valuable in identifying robust biomarkers for assessing labrum degeneration and calcification in synovial fluid or serum samples from hip OA patients. Secondly, tissue responses in the explant models were assessed in the absence of mechanical loading, which might be a more relevant factor than inflammatory stimuli in an *in vivo* setting. Thirdly, we could not assess sexual dimorphism in labrum tissues. Lastly, we have yet to evaluate whether conditioned medium from medium explants has a pro-osteogenic effect on joint-resident cells or explanted osteochondral tissues. Future studies should address these limitations. Furthermore, it would be interesting to investigate whether labrum calcification can be targeted through pharmacological means, such as the use of hydrogen sulfide donors [31], in the established explant model.

In conclusion, our study identified differential expression of VEGF and Pro-Col-Iα in acetabular labrum tissues from hip OA patients and fracture controls. Elevated levels of COMP, VEGF and Pro-Col-Iα were associated with labrum tissue calcification at the histological, though not detectable radiologically. This panel of protein markers holds promise as biomarkers for pre-radiographic detection of labrum calcification in hip synovial fluid. Explant models utilizing human joint tissues offer a promising approach to studying the molecular pathomechanisms underlying OA. Targeting TGF-beta signaling may offer a means to reduce vascular invasion and fibrosis in acetabular labrum tissue.

## Role of the funding source

This project was financially supported by the Swiss National Science Foundation (Grant number 190472) and Lausanne Orthopedic Research Foundation. The funders had no role in study design, data collection and analysis or drafting and submission of the manuscript.

## Declaration of competing interest

The authors declare that they have no conflicts of interest related to this study.

## Figures and Tables

**figs1:**
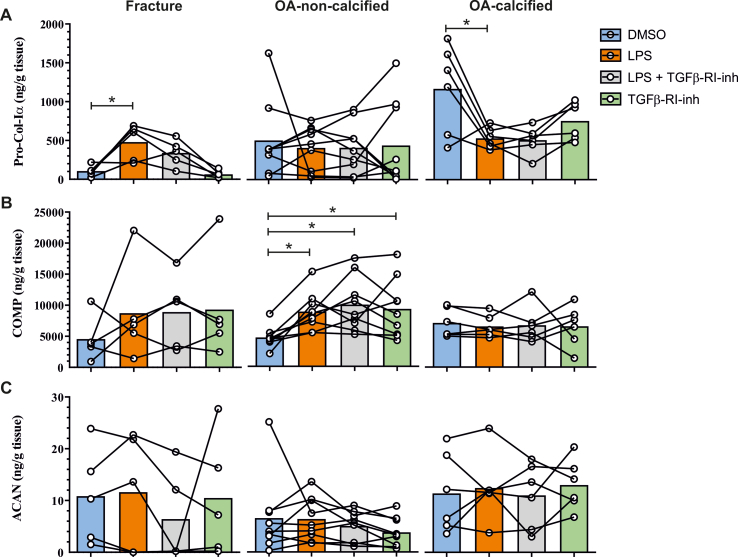


**figs2:**